# Acceptability and Attitude towards a Mobile-Based Home Exercise Program among Stroke Survivors and Caregivers: A Cross-Sectional Study

**DOI:** 10.1155/2019/5903106

**Published:** 2019-05-02

**Authors:** Amreen Mahmood, Vevita Blaizy, Aparajita Verma, Joel Stephen Sequeira, Dola Saha, Selvam Ramachandran, N. Manikandan, Bhaskaran Unnikrishnan, John M. Solomon

**Affiliations:** ^1^Department of Physiotherapy, School of Allied Health Sciences, Manipal Academy of Higher Education (MAHE), Manipal, Karnataka, India; ^2^Department of Health Information Management, School of Allied Health Sciences, Manipal Academy of Higher Education (MAHE), Manipal, Karnataka, India; ^3^Kasturba Medical College, Mangalore, Faculty of Health Sciences, Manipal Academy of Higher Education (MAHE), Manipal, India; ^4^Coordinator, Center for Comprehensive Stroke Rehabilitation and Research, Manipal Academy of Higher Education (MAHE), Manipal, India

## Abstract

**Background:**

Stroke is a leading cause of disability and requires continued care after hospital discharge. Mobile-based interventions are suitable to reduce the cost of stroke rehabilitation and facilitate self-management among stroke survivors. However, before attempting to use mobile-based home exercise program, it is crucial to recognize the readiness of stroke survivors and their caregivers to opt for such interventions.

**Objective:**

To assess the acceptability and attitude towards a mobile-based home exercise program among stroke survivors and their primary caregivers.

**Methods:**

A cross-sectional study was conducted among 102 participants to understand their attitude and acceptability towards mobile-based home exercise program. A validated 10-item questionnaire was adapted for the study. The questions which assessed the attitude were rated on a three-point Likert scale, with three denoting agree and one denoting disagree. The acceptability was assessed by their willingness to opt for a mobile-based home program services. A Chi-square analysis and cross-tabulation were performed to test differences between caregivers and patients. A logistic regression was performed to determine the effects of age, gender, and mobile phone on acceptability.

**Results:**

Ninety-two percent of caregivers and 90% of patients showed willingness to opt for mobile-based intervention. Majority of the participants showed a positive attitude towards this mode of treatment. There was no difference in the attitude noted among caregivers and patients (p>0.05) towards mobile-based intervention.

**Conclusion:**

The stroke survivors and caregivers welcomed the concept of mobile-based home exercise program even in a low-resource settings, but further studies to understand treatment and cost-effectiveness of this technology among the stroke survivors would lead to better implementation.

## 1. Introduction

### 1.1. Background

Stroke is one of the most disabling adult chronic diseases which requires the long-term continuance of exercises and physical activity [1, 2]. Home-based rehabilitation after stroke has growing evidence to optimize recovery [3]. Stroke patients receive instructions for home-based exercises at the time of discharge or during the follow-up visit. However, the amount of information, reinforcement, and support provided during hospital visits are limited [4].

Global Observatory for eHealth defines m-health or mobile health as “medical and public health practice supported by mobile devices, such as mobile phones, patient monitoring devices, personal digital assistants, and other wireless devices” [5]. M-health is an upcoming and innovative concept for improving interaction between healthcare providers, patients [6, 7], and researchers [8, 9]. M-health is found feasible and even showed significant outcomes in other health conditions such as diabetes and cardiac rehabilitation [10, 11]. Studies have reported that m-health was effective in reducing the risk factors for stroke [12]. However, the implementation effectiveness of m-health for rehabilitation after stroke is not yet established.

Though the last decade saw a significant upswing in the field of m-health, its maximum potential still lies unexplored [13]. With a growing user base of this technology, its potential is enormous and needs to be adequately tapped for best results. According to the International Telecommunication Union (ITU), there are now over 5 billion wireless subscribers, and over 70% of them reside in low- and middle-income countries and have access to the Internet even in remote areas [5]. India's telecommunication network is second largest in the world with 1.206 billion users as of September 2017 and has world's second-largest Internet user base [14]. This echoes the fact that time is ripe for considering m-health as a suitable intervention for improving health behavior after stroke in India and worldwide [15].

Mobile-based home exercises could be delivered in the form of video games for exercises [16], music software for gait training [17], or videos of exercises using mobile app [18]. The implementation of any intervention requires not only the effectiveness of an intervention but also high acceptability and sustainability [19]. Therefore, before attempting to incorporate m-health in home-based exercises after stroke, it is important to recognize the opinions and readiness of the stakeholders towards such intervention [20].

### 1.2. Objective

Therefore, we aimed to assess the acceptability and attitude of stroke survivors and primary caregivers towards a mobile-based home exercise program.

## 2. Material and Methods

### 2.1. Study Design

A cross-sectional questionnaire-based study was conducted among stroke survivors and primary caregivers of stroke patients.

### 2.2. Setting

The study was conducted in a tertiary care university teaching hospital of the semiurban region of coastal southern India, from August 2017 to March 2018. Necessary ethical approval was obtained from the Institutional Ethics Committee (IEC: 484/2017).

### 2.3. Participants

Adult stroke survivors who were medically stable, had functional communication abilities, and were able to comprehend either regional language (Kannada) or English were included in the study. The caregivers who primarily took care of stroke survivors and were fluent in either regional language or English were included. Stroke survivors with cognitive impairments and other associated neuromuscular disorders were excluded. Both stroke survivors and the caregivers were excluded if they did not use a mobile phone. We used purposive sampling to select the patients with stroke. All stroke survivors who were admitted and referred for motor rehabilitation or those who came for a follow-up visits during the study period were screened for eligibility criteria. The caregivers of the stroke survivors were recruited individually and not paired with the patients to avoid the bias of opinion. The participants were recruited into the study after obtaining their written informed consent.

### 2.4. Data Measurement

A validated questionnaire was adapted from our previous study which was aimed at exploring the acceptability and attitude of students towards m-health. This questionnaire was modified for stroke survivors focusing on home exercise program, e.g., items like ‘use of mobile phone application will help to do home exercises more independently' were added. Modifications in the questionnaire were agreed upon by all the authors and it was content validated by one expert from the field of Physiotherapy in Neurosciences and two experts from Health Information Management. Content validation was done for factors such as relevance, clarity, importance, and ethics of each item. The experts provided response between 1 and 5 on each question, where 1 indicated very low and 5 indicated very high for each factor. Questions which had 80% agreement between the experts were finally included in the questionnaire. Piloting of the questionnaire was done on one male and one female stroke survivors at two different time points to assess the administrative difficulties. The final questionnaire consisted of ten questions on attitude and acceptance towards m-health and its use for home-based exercises after stroke. The questions which assessed the attitude were rated on a three-point Likert scale, with three denoting agree and one denoting disagree. The acceptability was assessed by their willingness to opt for a mobile-based home exercise program and the charges that they were willing to pay for such services. The final questionnaire was translated to the regional language using parallel-back translation. The mobile-based home exercise program was developed for the stroke survivors and it consists of exercise prescription in the form of videos and audio instructions along with an educational video on stroke recovery and rehabilitation and feedback on everyday session. The exercise program model was shown to all the participants and they were asked to fill the questionnaire.

### 2.5. Study Size

The sample size was calculated with an expected proportion of 50% participants who are willing to accept a mobile-based home exercise program. Taking 10% as absolute precision and 7% nonresponse rate, the total sample size resulted in 102 participants.

### 2.6. Statistical Analysis

Descriptive statistics were used to analyze the data using Easy-R software. The scores obtained in the attitude questions were cross-tabulated to obtain percentages. The difference in the attitudes between the two groups was determined using Pearson's Chi-square test, where* P *<0.05 was considered to be statistically significant. Logistic regression was performed to ascertain the effect of age, gender, and type of phone used on acceptability and attitude towards mobile-based home exercise program.

## 3. Results

**Figure 1:**
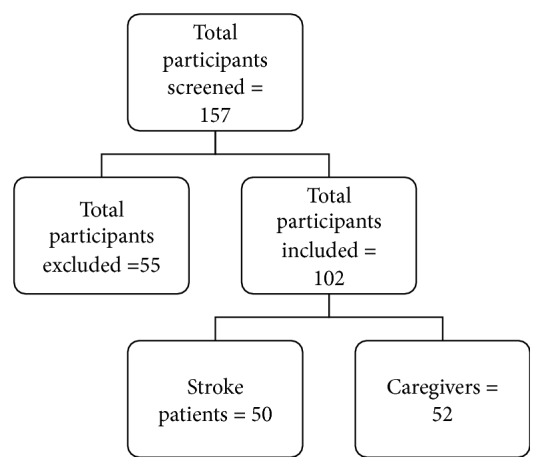
Flow of participants. **∗**Reasons for exclusion: unstable patients, aphasia, major comorbidities, declined to participate, and not using mobile phone.

**Figure 2:**
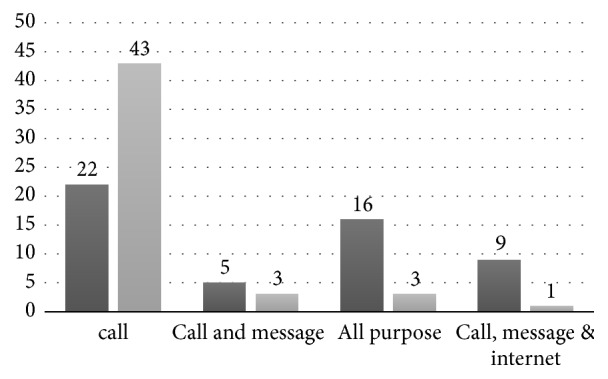
Most common uses of mobile phones among the participants. *∗*Dark grey: caregiver; light grey: patients.

We screened 157 potential participants for eligibility, 45 stroke patients were excluded as they were unstable or aphasic or had other major comorbidities. Ten caregivers were excluded as they declined to participate in the study or did not have a mobile phone.  Figure 1 shows the flow of participant recruitment. A total of 102 participants were included in the study, 50 stroke survivors with mean age of 55 ± 13 years, and 52 caregivers with mean age of 39 ± 12 years. The demographic details of the study participants are provided in Table 1. All the participants had experience of using mobile phones for more than one year except for one participant who started using mobile phone six months back.  Figure 2 represents the most common usage of mobiles among the participants. Four patients and 25 caregivers used Internet services on mobile phones. Most of the patients (96%) and caregivers (94.2%) had not used mobile phones for any healthcare services earlier.

### 3.1. Acceptability towards m-Health

The acceptability results revealed that majority of the patients (90%, n=45, 95% CI 0.81, 0.98) and caregivers (92.3%, n=48, 95% CI 0.85, 0.99) had the willingness to opt for mobile-based home exercise program after stroke (Table 3). Most of the stroke participants (72%, n=36) and caregivers (82.7%, n=43) showed readiness to pay nominal charges for such services. Only five patients (10%) and four caregivers (7.7%) were not willing to accept m-health. The reasons expressed by the participants for not opting this technology were using the phone less often and using it only for making calls and also the feeling that mobile phones are not beneficial for treatment purpose. Of the nine participants who did not opt for m-health technology, eight were basic mobile phone users, and one was a smartphone user, and all were of mean age 54 ±10 years.

### 3.2. Attitude towards m-Health

The attitude of stroke patients and caregivers towards mobile-based home exercises is shown in Table 2. The results indicated a high percentage of agreement towards these services; both among patients and caregivers. More than 80% of the participants accepted the benefits of mobile-based home exercise program, 72% agreed that it would lead to reduction of costs, and 70% believed that it will ensure confidentiality of information. Majority of the participants (82%-94.2%) perceived that mobile-based home exercise program would help in reduction of time in utilizing stroke care services; will be a useful reminder for home exercises; will improve awareness about stroke; will improve access to a therapist for follow-up; and make health information delivery faster.

### 3.3. Predictors of Acceptability and Attitude

The chi-square analysis showed no significant difference between caregivers and patients attitude (P>0.05) (Table 2). The result of logistic regression showed no difference on acceptance and attitude towards m-health between different age groups, gender, and type of phone used.

## 4. Discussion

This study reported the acceptance and attitude of stroke survivors and caregivers towards mobile-based home exercise program. It is important to know the acceptability because the intention to adopt a technology is determined by perceived usefulness, perceived effort, social influences, attitude towards technology, and mobile experience [21]. This study reflected the positive attitude and strong willingness of stroke survivors and caregivers towards mobile-based home exercises, which makes it a forthcoming medium to deliver home-based exercise program after stroke even in distant locations.

Some of the components, such as the use of mobile-based technology in cost reduction and in ensuring confidentiality and security of data were rated neutral by more than 15% of the participants. These responses reflect that many people were not sure about these aspects of m-health and its potential benefits to them. Hence it stresses the importance of educating patients and caregivers about the benefits of using mobile-based technology in healthcare and about safety measures followed for data protection. Participants who refused to opt for m-health were from middle to older age group who would have a fear of technology [22] and also the lack of exposure to smartphone devices would have influenced their decision. Moreover, the attributes vary based on subjective norms, perceived behavioral control, and attitudes [23]. The results of this study did not indicate any significant predictors of acceptability. Hence, there is a need to address the individualistic factors qualitatively in future.

Our findings are similar to a qualitative study conducted in sub-Saharan Africa, which showed a positive attitude towards using a mobile phone for seeking health services among caregivers of children, even without having a previous experience to the interactive-voice-response system [24]. A recent study assessed the attitude of stroke patients towards m-health technology living in a developed country and showed a positive attitude towards telecommunication for blood pressure control and medication adherence [25]. Exercises are more complex and require high self-efficacy to be independently performed by people with stroke [26]. This study revealed the inclination to opt for a mobile-based home exercise program after stroke. In contrast, another study conducted in Indian population reported low acceptability among patients and moderately positive attitude towards m-health among healthcare providers [27]. The difference in the findings could be because our questions were directed at using mobile-based home exercise tool as an adjunct and not as a replacement for direct contact with healthcare providers.

Acceptability is the most appropriate implementation outcome for individual-level analysis [19]. The results of this study showed that mobile-based home exercise program has high acceptability, which could lead to better implementation. This study explored the opinions of participants living in a low-income setting with lesser resources and infrastructure. Even in a semiurban region, the participants showed readiness for technology-based rehabilitation and welcome the unconventional concept of healthcare services. Additionally, this study included the perception of caregivers who are the cornerstone of the health care team and play a crucial role in implementing any lifestyle changes for a stroke patient. 

*Limitations.* Limitation of this study is the lack of stratification of participants based on income, education level, and duration of smartphones use that could have confounded the results. Another limitation is that the opinion of healthcare providers towards this technology was not included and it would have limited our understanding of implementing the mobile technology.

## 5. Conclusion

Stroke survivors and their caregivers showed remarkable acceptance and a positive attitude towards mobile-based home exercise program for stroke management even in a low-resource setting. Future studies should target treatment and cost-effectiveness of this technology in low-to-middle income countries. The results of this study can strengthen the prescription of mobile-based home exercise programs among healthcare providers and also help in improving its application for stroke survivors.

## Figures and Tables

**Table 1:** Demographic characteristics of the participants.

	*Patients* (n=50)	*Caregivers* (n=52)
*Age (years) *		
(Mean ±SD)	55.2 ± 13.39	39.78 ± 12.62
*Gender *		
Male	36 (72%)	20 (38%)
Female	14 (28%)	32 (62%)
*Type of phone used*		
Smart phone	10 (20%)	29 (56%)
Basic phone	40 (80%)	23 (44%)
*Duration of stroke (months)*		
Median (IQR)	0.45 (1.58)	

**Table 2:** Summary of responses for attitude towards mobile-based home exercise program after stroke.

Questions	Agree % (n)		Neutral % (n)		Disagree % (n)		p-value
	Patients	Caregivers	Patients	Caregivers	Patients	Caregivers	
Help in utilizing health care services for stroke by							
(a) Reducing costs	72.0 (36)	86.5 (45)	18.0 (9)	7.7 (4)	10.0 (5)	5.8 (3)	0.728
(b) Reducing time	88.0 (44)	90.4 (74)	10.0 (5)	9.6 (5)	2.0 (1)	0.0 (0)	0.32
(c) Convenience in visiting healthcare center	94.0 (47)	86.5 (45)	4.0 (2)	7.7 (4)	2.0 (1)	5.8 (3)	0.435

Beneficial in managing health after a stroke	88.0 (44)	84.6 (44)	8.0 (4)	11.5 (6)	4.0 (2)	3.8 (2)	0.906

Help to do home exercises independently after stroke	86.0 (43)	86.5 (45)	8.0 (4)	7.7 (4)	6.0 (3)	5.8 (3)	0.982

Reduce the need to visit therapist often	82.0 (41)	88.4 (46)	10.0 (5)	7.7 (4)	8.0 (4)	3.8 (2)	0.739

Reminder for home exercise sessions	92.0 (46)	92.3 (48)	4.0 (2)	7.7 (4)	4.0 (2)	0.0 (0)	0.384

Improve awareness about stroke management	84.0 (42)	94.2 (49)	10.0 (5)	5.8 (3)	6.0 (3)	0.0 (0)	0.237

Improving access to a therapist for follow-up/suggestions	86.0 (43)	94.2 (49)	12.0 (6)	5.8 (3)	2.0 (1)	0.0 (0)	0.415

Make health information delivery faster	82.0 (41)	82.7 (43)	18.0 (9)	15.4 (8)	0.0 (0)	1.9 (1)	0.785

Ensure confidentiality and security of information collected	70.0 (35)	80.8 (42)	26.0 (13)	19.2 (10)	4.0 (2)	0.0 (0)	0.367

Should be made available to all stroke patients	94.0 (47)	86.6 (45)	4.0 (2)	9.6 (5)	2.0 (1)	3.8 (2)	0.409

**Table 3:** Percentage acceptance towards mobile-based home exercise program among stroke survivors and caregivers.

Questions	Stroke survivors		Caregivers	
	Yes (%)	No (%)	Yes (%)	No (%)
Given a chance, would you opt for mobile-based home exercise program?	45 (90.0)	5 (10.00)	48 (92.3)	4 (7.70)

Would you opt for such services provided at nominal cost?	36 (72.0)	14 (28.0)	43 (82.7)	9 (17.3)

What charges are you willing to pay for such services?				
INR 250-500	31 (86.1)		38 (88.4)	
INR 500-750	4 (9.3)		3 (8.3)	
INR 750-1000	1 (2.3)		2 (5.6)	

*∗*INR=Indian Rupee.

## Data Availability

The data used to support the findings of this study are available from the corresponding author upon request.
